# The Molecular Determinants of Mitochondrial Membrane Contact With ER, Lysosomes and Peroxisomes in Neuronal Physiology and Pathology

**DOI:** 10.3389/fncel.2020.00194

**Published:** 2020-08-07

**Authors:** Yajin Liao, Yuan Dong, Jinbo Cheng

**Affiliations:** ^1^Center on Translational Neuroscience, College of Life & Environmental Science, Minzu University of China, Beijing, China; ^2^Department of Biochemistry, Medical College, Qingdao University, Qingdao, China

**Keywords:** mitochondria, membrane contact, determinant, physiological condition, pathological condition, neurological diseases

## Abstract

Membrane tethering is an important communication method for membrane-packaged organelles. Mitochondria are organelles with a bilayer membrane, and the membrane contact between mitochondria and other organelles is indispensable for maintaining cellular homeostasis. Increased levels of molecular determinants that mediate the membrane contact between mitochondria and other organelles, and their functions, have been revealed in recent years. In this review article, we aim to summarize the findings on the tethering between mitochondria and other organelles in physiological or pathological conditions, and discuss their roles in cellular homeostasis, neural activity, and neurodegenerative diseases.

## Introduction

Most of the organelles within cells are membrane-bound. Membrane tethering is one of the communication methods for signal/material exchanges between organelles. Membrane contact was first observed 60 years ago (Bernhard and Rouiller, 1956; Dalen et al., 1983; Grönblad and Akerman, 1984), however, the roles and molecular determinants of organelle juxtaposition were discovered only recently. The contact between two organelles is achieved by means of proteins that tether them directly. Mitochondria are vital ATP-generating organelles, and play a critical role in synapse activity, neurite outgrowth, neurogenesis, and neuronal cell death (Sun et al., 2013; Liao et al., 2015; Khacho et al., 2016; Norkett et al., 2016; Vaccaro et al., 2017). The dysfunction of mitochondria, including Ca^2+^ overload, excessive fission, disrupted distribution, and clearance of damaged mitochondria has been observed in multiple disorders of the nervous system (Calkins et al., 2011; Guardia-Laguarta et al., 2014; Lee et al., 2018; Berenguer-Escuder et al., 2020; Tsai et al., 2020). In previous studies, many advances have been made in the identification of the contacts among various organelles and on their structural determinants. These studies have proved that membrane contacts regulate several aspects of the biology and the behavior of mitochondria (Filadi et al., 2018; McLelland et al., 2018; Grossmann et al., 2019). Considering the example of mitofusin-1/2 (MFN1/2), their ability to control the endoplasmic reticulum (ER)-mitochondria tethering is associated with the activity of regulating the balance between mitochondrial fusion and fission (de Brito and Scorrano, 2008; Li et al., 2015; Qi et al., 2016). Either a decrease or an increase in the interaction levels between mitochondria and other organelles have been proved to induce mitochondrial dysfunction, in turn effecting energy metabolism, respiration, apoptosis, oxidative stress, and inflammation (De Vos et al., 2012; Lee et al., 2016). In addition, a growing amount of evidence indicates that the dysfunction of membrane contacts between mitochondria and other organelles is involved in the development of neural stem cell-related and neurodegenerative diseases, including Alzheimer’s disease (AD), Parkinson’s disease (PD), and amyotrophic lateral sclerosis (ALS; Area-Gomez et al., 2012; Lee et al., 2016, 2018; Stoica et al., 2016). In this review, we mainly aim to summarize the determinants of mitochondria contacts with other organelles under different physiological or pathological conditions.

## The Determinants of ER-Mitochondria Membrane Contact

### General Characteristics and Functions of ER-Mitochondria Membrane Contact

One of the most well-studied membrane contacts is ER-mitochondrial tethering. The interplay between these two organelles is necessary for the proper functioning of the cell, by maintaining Ca^2+^ homeostasis, lipid metabolism, and autophagy (Voss et al., 2012; Wideman et al., 2013; Gomez-Suaga et al., 2017b; Hirabayashi et al., 2017; Eisenberg-Bord et al., 2019).

**Figure 1:**
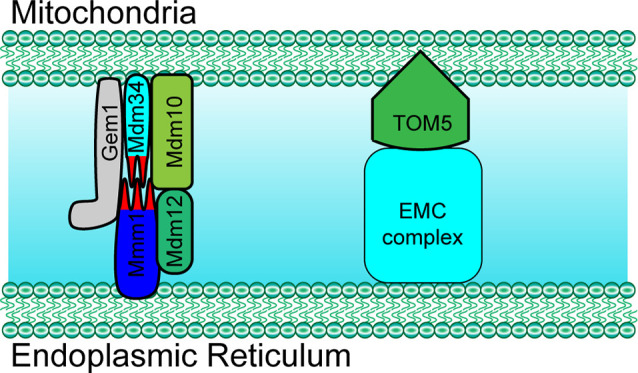
Paradigm of molecular determinants in endoplasmic reticulum (ER)-mitochondria tethering in yeast. Mdm10, Mdm34 and Gem1 located at the outer mitochondrial membrane (OMM) mediate ER-mitochondria tethering by forming a complex with ER membrane located proteins Mmm1 and cytosol Mdm12 in yeast. ER membrane protein complex (EMC) mediates ER-mitochondria contact by interacting with TOM5 at the mitochondrial OMM in yeast. Mdm10/34, mitochondrial distribution and morphology protein 10/34; Gem1, mitochondrial Rho GTPase 1; TOM5, translocase of outer membrane 5 kDa subunit.

The ER-mitochondria encounter structure (ERMES) is the first identified complex that was found to mediate the structural interaction between the ER and mitochondria. In yeast, the ERMES is composed of mitochondrial distribution and morphology protein 10/12/34 (Mdm10/12/34), maintenance of mitochondrial morphology protein 1 (Mmm1), and mitochondrial Rho GTPase 1 (Gem1; Figure 1; Kornmann et al., 2011; Jeong et al., 2016). Among these proteins, Mdm12, Mmm1, and Mdm34 contain a conserved synaptotagmin-like mitochondrial lipid-binding protein (SMP) domain that is important for the formation of the ERMES complex and to facilitate the contacts between the ER and mitochondria (AhYoung et al., 2015). Another conserved complex, the ER membrane protein complex (EMC), also plays an important role in tethering the ER to mitochondria (Lahiri et al., 2014). By interacting with the translocase of outer membrane 5 kDa subunit (TOM5) on the outer membrane of mitochondria, EMC1-6 of the EMC complex mediates the ER-mitochondria contact necessary for phosphatidylserine transfer in yeast (Figure 1; Lahiri et al., 2014). Importantly, both ERMES- and EMC-mediated tethering is essential for cell growth.

**Figure 2:**
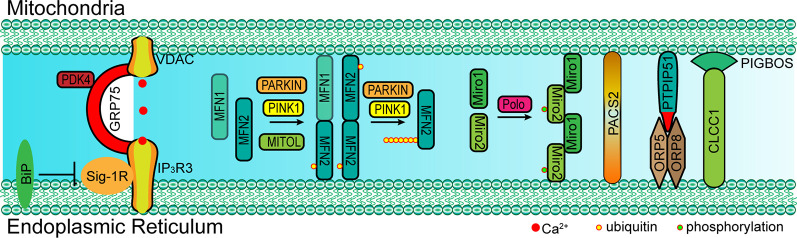
Paradigm of molecular determinants in ER-mitochondria tethering in mammal. Left: GRP75 interacts with both VDAC (located at OMM) and IP_3_R3 (located at MAM) to form a complex that mediates the ER-mitochondria contact. The interaction between Sig-1R and IP_3_R3 promotes the stability of IP_3_R3, which is essential for the IP_3_R3-GRP75-VDAC complex, while BiP inhibits this interaction by competitive binding to Sig-1R. Middle: MFN1/2 form a dimer at MAM and mediate ER-mitochondria tethering. PARKIN, PINK1 and MITOL mediate the mono-ubiquitination of MFN2 and promote MFN2 translocation to MAM. However, the poly-ubiquitination of MFN2 mediated *via* PARKIN and PINK1 leads to the tethering disruption. Right: Miro1/2 form a cluster at MAM and mediate ER-mitochondria tethering, and the phosphorylation of Miro2 by polo kinase is essential for the formation of the Miro cluster. Cytosolic PACS2 recruits to the MAMs and OMM to mediate ER-mitochondria membrane contact. The OMM-resident PTPIP51 interacts with ORP5/8 at MAM to maintain mitochondrial morphology and respiratory functions. The OMM-resident PIGBOS interacts with the ER membrane protein CLCC1 to maintain ER–mitochondrial contact. GRP75, 75 kDa glucose-regulated protein; VDAC1/2, voltage-dependent anion-selective channel protein; IP_3_R3, inositol 1,4,5-trisphosphate receptor type 3; MFN1/2, mitofusin-1/2; Sig-1R, sigma-1 receptor; ORP5/8, oxysterol-binding protein (OSBP)-related proteins 5/8; PTPIP51, protein tyrosine phosphatase interacting protein-51; CLCC1, chloride channel CLIC-like protein 1; PIGBOS, PIGB opposite strand 1; PACS2, phosphofurin acidic cluster sorting protein 2; Miro1/2, mitochondrial Rho 1/2; MITOL: mitochondrial ubiquitin ligase; PINK1, PTEN-induced putative kinase protein 1.

In mammal cells, some proteins also localize into a subcompartment on the membrane of the ER, which makes contact with mitochondria and is referred to as the mitochondria-associated ER membrane (MAM; Vance, 1990; Rusinol et al., 1994; Csordás et al., 2006; Area-Gomez et al., 2012). MAM molecular determinants are dynamic and shape ER-mitochondria function (Poston et al., 2013; Gelmetti et al., 2017; Ma et al., 2017; Pera et al., 2017). The classic molecules that are involved in ER-mitochondria tethering include MFN1/2, phosphofurin acidic cluster sorting protein 2 (PACS2), sigma-1 receptor (Sig-1R), F-box protein FBXL2 (FBXL2), 75 kDa glucose-regulated protein (GRP75), voltage-dependent anion-selective channel protein 1/2 (VDAC1/2), and inositol 1,4,5-trisphosphate receptor type 3 (IP_3_R3; Figure 2 and Table 1; Simmen et al., 2005; Szabadkai et al., 2006; Hayashi and Su, 2007; de Brito and Scorrano, 2008; Mori et al., 2013; Naon et al., 2016; Kuchay et al., 2017; Veeresh et al., 2019). Both the formation of the MFN2 dimer and IP_3_R3-VDAC-GRP75 complex in the MAM could mediate the physical linkage between the ER and mitochondria (Csordás et al., 2006; Szabadkai et al., 2006; de Brito and Scorrano, 2008; McLelland et al., 2018). In addition, some molecules have been proven to enhance ER-mitochondria contacts in a dose-dependent manner, which promote the transfer of Ca^2+^ and other chemicals between the ER and mitochondria (Chen et al., 2012; Mori et al., 2013; McLelland et al., 2018; Yu et al., 2019). Furthermore, accumulative evidence suggests that the post-translational modification of these proteins is also essential for the tethering activity and dynamic balance of ER-mitochondria contacts involving the aforementioned determinants (McLelland et al., 2018). Considering the example of MFN2, a mitochondrial outer membrane protein that mediates fusion, mitophagy regulates ER-mitochondria contacts (de Brito and Scorrano, 2008; Chen et al., 2012; Naon et al., 2016). MFN2 ablation or silencing enhances ER-mitochondria close contacts and leads cells more sensitive for mitochondrial calcium overload-dependent cell death (Filadi et al., 2015). Moreover, MFN2-mediated ER-mitochondria tethering is regulated by ubiquitination. Mitochondrial ubiquitin ligase (MITOL)-mediated ubiquitination, PTEN-induced putative kinase protein 1 (PINK1) and Parkin-mediated mono-ubiquitination of MFN2 promote its tethering activity, while PINK1 and Parkin-mediated poly-ubiquitination of MFN2 result in MFN2 retrotranslocation and disrupt ER-mitochondria contacts to drive mitophagy (Figure 2; Sugiura et al., 2013; Basso et al., 2018; McLelland et al., 2018). These post-translational modifications likely explain the different findings on the role of MFN2 in keeping mitochondria and ER together (Naon et al., 2016; Filadi et al., 2017). Besides PINK1 and Parkin, other mitophagy-associated proteins, such as Beclin-1, have been found to relocalize in the MAMs during mitophagy (Gelmetti et al., 2017; McLelland et al., 2018). The relocalization of these proteins in the MAMs promotes ER-mitochondrial tethering and autophagosome formation (Gelmetti et al., 2017). Apart from post-translational modification, the interaction between the MAM components and other proteins also affects the ER-mitochondria contacts. Considering the example of IP_3_R3-GRP75-VDAC complex, the IP_3_R3-PTEN interaction and IP_3_R3-Sig-1R interaction promote the stability of IP_3_R3 and maintain the complex, while the IP_3_R3-BiP interaction and IP_3_R3-FBXL2 interaction induce the degradation of IP_3_R3 and disruption of the complex (Hayashi and Su, 2007; Kuchay et al., 2017). The Transglutaminase type 2 (TG2) was found to interact with GRP75 and to subsequently decrease the interaction level between GRP75 and IP_3_R3, thereby maintaining mitochondrial Ca^2+^ homeostasis. The loss of TG2 was found to increase GRP75-IP_3_R3 interaction levels and to decrease ER-mitochondria contact levels as well as mitochondrial Ca^2+^ levels (D’Eletto et al., 2018). Pyruvate dehydrogenase kinase 4 (PDK4) is another GRP75-interacting protein, and the upregulated expression of PDK4 could promote the formation of the VDAC1-GRP75-IP_3_R3 complex, which results in increased ER-mitochondria contact levels (Thoudam et al., 2019).

**Table 1:** The molecular determinant for endoplasmic reticulum (ER)-mitochondria tethering in mammal cell.

Tethering complex	Localization	Function	Pathogenicity
IP_3_R3-GRP75-VDAC, Sig-1R, BiP, TOM70, TG2, PDK4, FBXL2, Aβ	ER membrane and MAMs: IP_3_R3, Sig-1R, BiP (Hayashi and Su, 2007) Mitochondrial OMM: VDAC, TOM70 (Shoshan-Barmatz et al., 2008; Filadi et al., 2018) Cytosol: TG2, PDK4, FBXL2, GRP75 (D’Eletto et al., 2018; Thoudam et al., 2019)	1. IP_3_R3-GRP75-VDAC complex mediates ER-mitochondria Ca^2+^ transfer and homeostasis (Szabadkai et al., 2006). 2. Sig-1R, PDK4, TOM70 and Aβ promote the formation of IP_3_R3-GRP75-VDAC complex (Filadi et al., 2018; Thoudam et al., 2019). 3. TG2, BiP and FBXL2 inhibit the complex formation (Hayashi and Su, 2007; D’Eletto et al., 2018).	1. Upregulated IP3R3-GRP75-VDAC complex is associated with mitochondrial Ca^2+^ overload (Kuchay et al., 2017). 2. Mutations in Sig-1R are associated with ALS and neuropathy (Al-Saif et al., 2011; Gregianin et al., 2016). 3. Aβ-induced upregulation of IP_3_R3 and VDAC are associated with AD (Hedskog et al., 2013).
MFN1/2 dimer, PINK1, PARKIN, BECN1, MITOL	Mitochondrial OMM and MAMs: MFN1/2 Cytosol: PINK1, PARKIN, BECN1, MITOL (Sugiura et al., 2013; Basso et al., 2018; McLelland et al., 2018)	1. MFN1/2 dimer mediates ER-mitochondria Ca^2+^ transfer and mitophagy (de Brito and Scorrano, 2008; Chen et al., 2012; Qi et al., 2016). 2. PINK1, PARKIN and BECN1 mediated ubiquitination of MFN2 is essential for MFN2 translocation.	Cells lacking MFN1/2 are associated with mitochondrial fragmentation (Chen et al., 2003).
PIGBOS-CLCC1	ER membrane: CLCC1 Mitochondrial OMM: PIGBOS (Chu et al., 2019)	PIGBOS mediates the ER-mitochondrial tethering by interacting with an identified protein (Chu et al., 2019).	Loss of PIGBOS elevated unfolded protein response (UPR) and increased cell death under stress conditions (Chu et al., 2019).
Miro1/2 cluster, Polo	Mitochondrial OMM and MAMs (Lee et al., 2016; Modi et al., 2019)	1. Miro1/2 cluster mediates Mitochondrial Ca^2+^ uptake (Modi et al., 2019). 2. Polo mediated phosphorylation of Miro promotes the activation of Miro (Lee et al., 2016).	Cells lacking Miro leads to decreased mitochondrial Ca^2+^ uptake, while overexpression of Miro1/2 result in Ca^2+^ overload and cell death (Lee et al., 2016; Modi et al., 2019).
VAPB-PTPIP51, FUS, TDP43	ER membrane and MAMs: VAPB Mitochondrial OMM: PTPIP51 (De Vos et al., 2012) Cytosol: FUS, TDP43 (Stoica et al., 2014, 2016)	VAPB-PTPIP51 interaction mediates ER-mitochondria tethering, which is essential for mitochondrial Ca^2+^ homeostasis, mitophagy and neural activity (De Vos et al., 2012; Gomez-Suaga et al., 2017b; Gómez-Suaga et al., 2019).	1. Loss of VAPB or PTPIP51 results in decreased synaptic activity (Gómez-Suaga et al., 2019). 2. Disrupted VAPB-PTPIP51 interaction and loss of function of the complex is associated with ALS (Stoica et al., 2014, 2016).
ORP5/8-PTPIP51	ER membrane and MAMs: ORP5/8 (Galmes et al., 2016) Mitochondrial OMM: PTPIP51	ORP5/8-PTPIP51 interaction mediated ER-mitochondria tethering is associated with mitochondrial morphology and respiratory function (Galmes et al., 2016).	Cells lacking ORP5 display low respiratory function (Galmes et al., 2016).
PACS2	mitochondria and MAMs (Yu et al., 2019)	ER-mitochondrial Ca^2+^ transfer and apoptosis (Yu et al., 2019).	Downregulation of PACS2 leads to decreased ER-mitochondria tethering and cells become more sensitivity to ox-LDL-induced cell death (Yu et al., 2019).
Rmdn3	Mitochondrial OMM (Fecher et al., 2019)	Rmdn3 mediates the ER-mitochondria tethering in Purkinje cells (Fecher et al., 2019).	Cells lacking Rmdn3 leads to decreased autophagosome formation (Gomez-Suaga et al., 2017a).
PDZD8	ER membrane (Hirabayashi et al., 2017)	PDZD8 mediates the dendritic ER-mitochondria Ca^2+^ transfer in neuron upon stimulation (Hirabayashi et al., 2017).	PDZD8-deficiency leads to decreased ER-mitochondrial Ca^2+^ release-uptake coupling (Hirabayashi et al., 2017).

Besides these canonical molecular determinants that mediate ER-mitochondria tethering, in the last few years, several other proteins that translocate to the MAM and that mediate ER-mitochondria tethering have been identified [i.e., Mitochondrial Rho (Miro) and PIGB opposite strand 1 (PIGBOS); Chu et al., 2019; Modi et al., 2019]. In 2016, Miro1 and Miro2, two mitochondrial Rho GTPases, were discovered to mediate ER-mitochondria tethering (Lee et al., 2016). Recent studies have revealed that Miro1 and Miro2 form nanometer-sized clusters along the outer membrane of the mitochondria and are components of the mitochondrial contact site and cristae organizing system (MICOS) and ER-mitochondria contact sites (ERMCS; Figure 2; Lee et al., 2016; Modi et al., 2019). The loss of Miro1/2 results in decreased ER-mitochondria contact sites and mitochondrial Ca^2+^ uptake (Figure 1; Modi et al., 2019). Another study has indicated that Miro can be phosphorylated by polo-like kinase 1, leading to the activation of Miro (Lee et al., 2016). In neural stem cells, the inactivation of Miro leads to the depletion of mitochondrial Ca^2+^ levels resulting in a metabolic impairment, and the overexpression of Miro1/2 results in Ca^2+^ overload and cell death (Lee et al., 2016). PIGBOS, a novel microprotein that localizes on the outer membrane of the mitochondria (Chu et al., 2019), is found to mediate ER-mitochondria contact. The loss of PIGBOS elevates unfolded protein response (UPR) and increases cell death under stress conditions. The results of further investigation indicate that PIGBOS is able to interact with chloride channel CLIC-like protein 1 (CLCC1) on the membrane of the ER, and this interaction is essential for the function of PIGBOS (Figure 2; Chu et al., 2019). However, the interaction between PIGBOS and CLCC1 does not act as a tether between the ER and mitochondria (Chu et al., 2019). The oxysterol-binding protein (OSBP)-related proteins 5/8 (ORP5/8) mediate ER-mitochondria tethering by interacting with protein tyrosine phosphatase interacting protein-51 (PTPIP51; Figure 2). The depletion of ORP5/8 results in changes in the mitochondrial morphology and respiratory functions (Galmes et al., 2016). As the most extensively studied type of membrane contact, an increasing number of determinants that are involved in ER-mitochondria tethering is being discovered (Table 1), which promotes the understanding of the functions and regulatory mechanisms of ER-mitochondria contacts.

## The Functions of ER-Mitochondria Membrane Contact in the Nervous System

**Figure 3:**
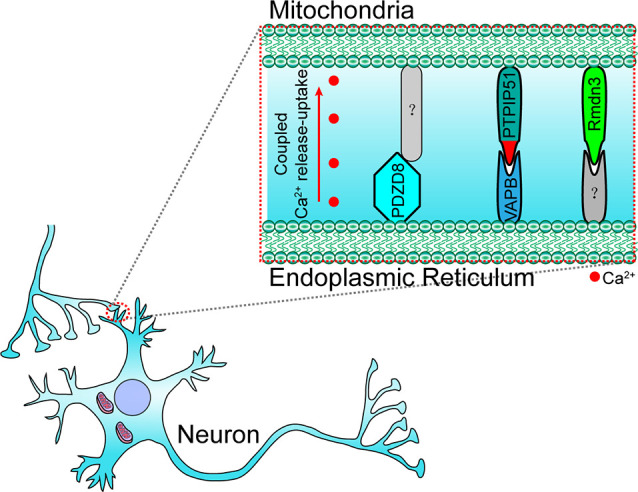
Paradigm of molecular determinants in ER-mitochondria tethering in the dendrite of neurons. In the dendrite, VAPB (located at ER) interacts with PTPIP51 (located at OMM) to maintain ER–mitochondria tethering and synaptic activity-coupled Ca^2+^ release-uptake. PDZD8 (located at ER) and Rmdn3 (located at OMM) are also involved in synaptic activity-coupled Ca^2+^ release-uptake and ER-mitochondria membrane contact. However, their interacting partners have still not been identified. VAPB, vesicle-associated membrane protein-associated protein B; PDZD8, PDZ domain-containing protein 8; Rmdn3, regulator of microtubule dynamics protein 3.

In neuronal cells, some molecules that mediate the local ER-mitochondria contacts regulate dendritic Ca^2+^ homeostasis in order to facilitate the adaptation to synapse stimulation, thereby playing a critical role in synaptic integration properties and plasticity. For example, vesicle-associated membrane protein-associated protein B (VAPB) and PTPIP51 form a complex at the synapses and mediate ER-mitochondria tethering (Figure 3; Gómez-Suaga et al., 2019). The interaction between VAPB (localization in the ER) and PTPIP51 (localization in the mitochondria) is critical for mitochondrial Ca^2+^ homeostasis (De Vos et al., 2012). The loss of VAPB or PTPIP51 results in higher cytosolic Ca^2+^ levels in the case of dendrite postsynaptic stimulation. In addition, the number of dendritic spines is reduced in VAPB- or PTPIP51-silenced neurons (Gómez-Suaga et al., 2019). Recently, on performing cell-type-specific profiling of the brain mitochondria, a regulator of microtubule dynamics protein 3 (Rmdn3) was found to predominantly mediate ER-mitochondria tethering in the Purkinje cells (Figure 3), rather than in the astrocyte and granule cells (Fecher et al., 2019). Even if a previous study suggests that Rmdn3 mediates the ER-mitochondria tethering by interacting with VAPB, the mechanism of Rmdn3-mediated ER-mitochondria tethering in neurons is unknown (Gomez-Suaga et al., 2017a). Recently, PDZ domain-containing protein 8 (PDZD8), a SMP domain-containing protein localized in the ER membrane, has been proven to mediate ER-mitochondria tethering in the neurons (Figure 3), which is critical for dendrites activity (Hirabayashi et al., 2017). Usually, Ca^2+^ released from the ER can induce the subsequent mitochondrial Ca^2+^ uptake (Hirabayashi et al., 2017). However, the mitochondrial Ca^2+^ import is significantly reduced in PDZD8-deficient neurons, resulting in higher Ca^2+^ levels in the dendrites (Hirabayashi et al., 2017). These results suggest that particular determinants mediate the ER-mitochondria membrane contacts in the neurons, especially in the neural dendrites, and that the absence of these proteins could result in decreased synaptic plasticity and activity.

**Figure 4:**
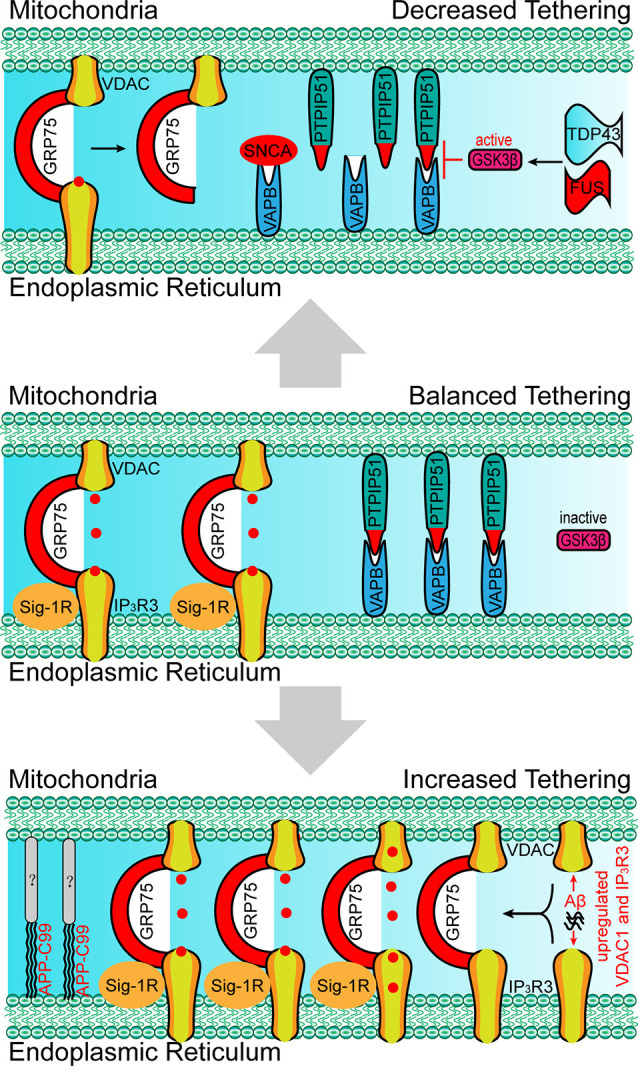
Paradigm of changes in molecular determinants and ER-mitochondria tethering in neurodegenerative disorders. Upper: the absence of Sig-1R at MAM leads to the de-stabilization of IP_3_R3, resulting in the decreased IP_3_R3-GRP75-VDAC complex and ER-mitochondria tethering, which is associated with the development of amyotrophic lateral sclerosis (ALS). SNCA, FUS and TDP43 disrupt the VAPB-PIPTP51 interaction and ER–mitochondria contact, which is associated with the development of Parkinson’s disease (PD) and ALS. Bottom: the accumulation of Aβ at MAM promotes the expression of VDAC and IP_3_R3, which leads to the increased formation of the IP_3_R3-GRP75-VDAC complex, ER-mitochondria tethering, and mitochondrial Ca^2+^ overload. APP-C99 is also observed at MAMs, which enhances the ER-mitochondrial contact. The presence of Aβ and APP-C99 at MAMs are associated with the development of Alzheimer’s disease (AD). SNCA, synuclein-α; TDP43, TAR DNA-binding protein 43; FUS, RNA-binding protein FUS; Aβ, β amyloid.

Besides the molecules that are present within the EMRES or MAM under normal physiological conditions, some molecules (i.e., APP-C99 and Tau) are found to localize within the ERMES or MAM under pathological conditions, resulting in excessive ER-mitochondria contacts, and mitochondrial Ca^2+^ overload, and cell death (Guardia-Laguarta et al., 2014; Pera et al., 2017; Cieri et al., 2018). Furthermore, excessive ER-mitochondria contacts have been recently found to be involved in neurodegenerative diseases. Based on the findings through biopsies of AD patients, the number of ER-mitochondria contact sites is positively correlated with elevated ventricular cerebrospinal fluid β-amyloid (Aβ) levels (Leal et al., 2018). A nanomolar concentration of Aβ is sufficient to increase the expression of VDAC1 and IP_3_R3, resulting in elevated ER-mitochondria contact levels and mitochondrial Ca^2+^ overload (Figure 4; Hedskog et al., 2013; Schreiner et al., 2015). The amyloid-beta precursor protein (APP)-C99, the C-terminal fragment of APP, is also found to localize in the MAM (Pera et al., 2017). The accumulation of C99 in the MAM region induces sphingolipid turnover (thereby leading to altered lipid composition in both the MAM and mitochondrial membrane), increased ER–mitochondria contacts and mitochondrial dysfunction (Figure 4; Pera et al., 2017), indicating that C99 may act as a risk factor for AD. Tau, another risk factor for AD and other types of dementia, is found to be present in the outer membrane of mitochondria and in the mitochondrial intermembrane space. Tau localization at mitochondria affects their distribution and enhances Ca^2+^ transfer from the ER to mitochondria (Cieri et al., 2018). Moreover, dysregulated ER-mitochondria contacts have also been observed in PD models. Synuclein-α (SNCA), a critical protein for the development of PD, has been proven to enhance the ER-mitochondria contacts, resulting in mitochondrial Ca^2+^ overload (Guardia-Laguarta et al., 2014; Calì et al., 2019). In neurons that were differentiated from PD-patient derived induced pluripotent stem cells (iPSC) with mutations in Parkin and PINK1, the ER-mitochondria contacts were found to be increased, resulting in elevated ER to mitochondrial lipid trafficking and the disrupted production of neuropeptide-containing vesicles (Valadas et al., 2018). Increased ER-mitochondria contacts and mitochondrial Ca^2+^ levels induced by PINK1 mutations lead to mitochondrial enlargement and neuronal cell death. These phenotypes could be rescued by the inhibition of Miro or components of the ERMES (Valadas et al., 2018).

In conclusion, these results suggest that some molecules could enhance ER-mitochondria tethering by acting as membrane contact determinants or enhancers under pathological conditions, which leads to mitochondrial Ca^2+^ overload, oxidative stress, neuroinflammation, or apoptosis.

Apart from excessive ER-mitochondria contacts being associated with the development of diseases, decreased ER-mitochondria contacts are also found to induce the onset of abnormalities in cellular metabolism, especially in the neurons. For example, MITOL-mediated ubiquitination of MFN2 is critical for the formation of the MFN2 dimers and in facilitating the tethering of ER-mitochondria (Sugiura et al., 2013). The loss of MITOL leads to a reduction in ER-mitochondria contact sites and an increase in oxidative stress in the neurons (Nagashima et al., 2019), which also render cells more vulnerable to ER-stress induced apoptosis (Takeda et al., 2019). The mutation of Miro1 in PD patients leads to decreased ER-mitochondria contacts and low basal mitochondrial Ca^2+^ levels, thereby impairing energy metabolism and mitophagy (Grossmann et al., 2019). In VAPB-, PTPIP51-, or PDZD8-deficient neurons the lack of ER-mitochondria tethering at the synapses lead to decreased synaptic activities (Hirabayashi et al., 2017; Gómez-Suaga et al., 2019). Moreover, the loss of VAPB/PTPIP51-mediated ER-mitochondria contact causes a reduced number of dendritic spines in the neurons (Gómez-Suaga et al., 2019). In familial PD involving mutations in SNCA, mutated SNCA interacts with VAPB and decreases the VAPB-PTPIP51 interaction, leading to decreased dendritic ER-mitochondria contact and low synaptic activity levels (Figure 4; Paillusson et al., 2017). In addition, shrunken mitochondria were observed in neurons with mutated SNCA neurons, indicating morphological changes in the mitochondria (Little et al., 2018). Apart from SNCA mutants, the RNA-binding protein FUS can also disrupt the VAPB-PIPTP51 interaction (Figure 4). A reduction in mitochondrial adenosine triphosphate (ATP) production and Ca^2+^ concentration is observed in FUS-upregulated neurons, which may be associated with the development of ALS and frontotemporal dementia (FTD; Stoica et al., 2016). TAR DNA-binding protein 43 (TDP43), another ALS-associated protein, can also inhibit the VAPB-PIPTP51 interaction (Figure 4). Cells with high expression levels of TDP43 were found to exhibit the same characteristics as those of FUS-upregulated cells, including increased cytosolic Ca^2+^ levels and decreased mitochondrial Ca^2+^ levels (Figure 3; Stoica et al., 2014). In addition, the inhibition of the VAPB-PTPIP51 interaction mediated by FUS and TDP43 is associated with the activation of glycogen synthase kinase-3 beta (Stoica et al., 2014, 2016). Decreased ER-mitochondria tethering is also found in Sig-1R- and SOD1-linked ALS (Watanabe et al., 2016). Sig-1R locates in the MAM and promotes ER-mitochondria tethering by stabilizing IP3R3 (Hayashi and Su, 2007). Aberrant subcellular distribution of Sig-1R mutations is associated with ALS and distal hereditary motor neuropathy (Al-Saif et al., 2011; Almendra et al., 2018). Further studies reveal that aberrant subcellular distribution of Sig-1R results in the decrease of ER-mitochondria tethering and mitochondrial Ca^2+^ levels (Figure 4; Al-Saif et al., 2011; Gregianin et al., 2016). In addition, increasing ER-mitochondria tethering by activation of Sig-1R is proven to be beneficial for SOD1-linked ALS. Considered together, the above studies indicate that decreased ER-mitochondria contacts or protein interaction changes are also harmful and result in alterations in cell fate, neural activity, and synaptic plasticity in the neurons.

In summary, apart from the classic membrane contact determinants that mediate the ER-mitochondria communication and metabolism, some disease-associated abnormal proteins or peptides could also play critical roles as determinants or regulators that disrupt the contact balance between the ER and mitochondria. Enhanced ER-mitochondria contact levels in the soma of neurons induce mitochondria Ca^2+^ overload and neuronal cell death, while decreased ER-mitochondria contact levels in the dendrites of neurons lead to reduced synaptic activity and spine density, suggesting that dysregulated ER-mitochondria interaction are involved in the development of some neurodegenerative disorders, such as AD, PD and ALS.

## The Determinants of Mitochondria-Lysosome Membrane Contact and Mitochondria-Peroxisome Membrane Contact

### Mitochondria-Lysosomes Membrane Contacts

Besides mitochondria-ER contacts, determinants of mitochondria-lysosomes and of mitochondria-peroxisomes contacts have been recently discovered, further enhancing the understanding of the communication between these organelles in the cells. Here, we have mainly summarized the determinants between these organs in the neuronal system.

A lysosome is a highly dynamic organelle that mediates the degradation of unfolded proteins, damaged organelles, and pathogen-derived components. Under stress conditions or during the process of aging, the lysosome-mediated clearance of damaged mitochondria is crucial for maintaining mitochondrial homeostasis (Wang and Klionsky, 2011). The accumulation of damaged mitochondria is observed in multiple cells with dysfunction in lysosome performance, which is also involved in development neurodegenerative diseases. However, because the lysosome-mediated clearance of damaged mitochondria is believed to be autophagy-dependent, the mitochondrial-lysosome interaction has been considered indirect membrane contact.

Recently, direct mitochondria-lysosome contacts have been discovered through multiple super-resolution imaging modalities. Ras-related protein Rab-7 (RAB7) was the first identified mitochondrial-lysosome membrane contact determinant (Wong et al., 2018). The presence of RAB7 is sufficient to mediate mitochondrial-lysosome tethering. The recruitment of TBC1 domain family member 15 (TBC1D15) by mitochondrial fission 1 protein (FIS1) at the membrane of mitochondria, enhances RAB7 activity (GTP hydrolysis), which results in mitochondrial-lysosome untethering (Wong et al., 2018). The GTP- guanosine diphosphate (GDP) cycling dynamic regulates the mitochondrial-lysosome contact and fission of the mitochondria. Moreover, a deficiency in *RAB7* levels in the neurons causes axonal degeneration and the development of Charcot-Marie-Tooth neuropathy type 2B (Ponomareva et al., 2016). With developments in imaging technology, apart from RAB7, we believe that other molecules mediating and controlling mitochondrial-lysosome tethering will be identified and characterized in the future.

### Mitochondria-Peroxisomes Membrane Contacts

Peroxisomes are membrane-bound oxidative organelles that play an indispensable role in the metabolism of fatty acids, glyoxylate, amino acids, and reactive oxygen species (ROS; Schrader et al., 2015). The evidence generated by studies on yeast demonstrate that peroxisomes are localized to mitochondria-ER junctions and sites of acetyl-CoA synthesis (Cohen et al., 2014). This finding suggests that the mitochondria-peroxisome contacts may be important for the β-oxidation of fatty acids in the peroxisomes or mitochondria. In mammal cells, mitochondria-derived vesicles (MDVs) are considered as one of the origins of peroxisomes, as some of the components of mitochondria are selectively integrated into peroxisomes (Sugiura et al., 2017). Peroxisomal membrane protein 11 (Pex11), a peroxin that is localized within the peroxisomes, is found to interact with Mdm34 (localized in the membrane of the ER and one of the components of the ERMES) in yeast (Mattiazzi Ušaj et al., 2015). In addition, the initiation of membrane contact between peroxisomes and mitochondria mediated by Pex11 and Mdm34 is ER-dependent (Mattiazzi Ušaj et al., 2015). On systematically mapping the contact sites, Pex34 and Fzo1 were discovered to localize at the membrane of peroxisomes, which can facilitate their tethering to mitochondria. The loss of Pex34 results in reduced β-oxidation of fatty acids (Shai et al., 2018). Acyl-coenzyme A-binding domain 2 (ACBD2)/ECI2 isoform A is another newly identified protein, with a C-terminal peroxisome targeting signal-1, which aids in the localization of ACBD2/ECI2 in peroxisomes. Further, by interacting with TOM20, ACBD2/ECI2 can mediate membrane contact between peroxisomes and mitochondria (Fan et al., 2016). A functional study revealed that ACB2/ECI2-TOM20 mediated peroxisome-mitochondria tethering is essential for the biosynthesis of steroids in Leydig Cells (Fan et al., 2016). Considered together, these studies suggest that proteins localized at the membrane of mitochondria and peroxisomes regulate the mitochondria-peroxisome membrane contacts and affect the lipid metabolism in peroxisomes.

In the nervous system, although the mitochondria-lysosome membrane contact and mitochondria-peroxisome membrane contact have not been studied as much as the mitochondria-ER contact, we believe that more and more contact determinants will be discovered and characterized in the near future.

## Concluding Remarks

Mitochondria exchange signals and materials with other organelles through membrane contacts. During this process, molecular determinants dynamically regulate the tethering and untethering between mitochondria and other organelles. However, in pathological conditions, some disease-associated molecules have been retrieved at MAM, where they act as membrane contact determinants (such as Aβ), disrupt physiological interactions *via* post-translational modification (such as PINK1), and participate in competitive interactions (such as FUS; Figure 1). These abnormal proteins present in the MAM cause dysfunctions in the tethering between mitochondria and other organelles, and thus leads to subsequent mitochondrial Ca^2+^ overload, lower synaptic activity levels, metabolic disorders, and finally neural cell death. An increasing amount of evidence shows that the dysregulation of mitochondria membrane contacts with other organelles cause mitochondrial dysfunctions underlying neurodegenerative diseases. However, extensive investigations are still needed to fully characterize the regulatory mechanism involved. Accordingly, in clinical practice, pharmacological modulation of contacts still has a long way to go.

## Author Contributions

YL conceived the review topic, reviewed the literature, wrote the manuscript, and prepared the figure. YD and JC reviewed the manuscript. YL and JC performed a comprehensive review of the literature.

## Conflict of Interest

The authors declare that the research was conducted in the absence of any commercial or financial relationships that could be construed as a potential conflict of interest.

## Abbreviations

MFN1/2: mitofusin-1/2
ER: endoplasmic reticulum
AD: Alzheimer’s disease
PD: Parkinson’s disease
ALS: amyotrophic lateral sclerosis
ERMES: ER-mitochondria encounter structure
Mdm10/12/34: mitochondrial distribution and morphology protein 10/12/34
Mmm1: maintenance of mitochondrial morphology protein 1
Gem1: mitochondrial Rho GTPase 1
SMP: mitochondrial lipid-binding protein
EMC: ER membrane protein complex
TOM5: translocase of outer membrane 5 kDa subunit
MAM: mitochondria-associated ER membrane
PACS2: phosphofurin acidic cluster sorting protein 2
GRP75: 75 kDa glucose-regulated protein
VDAC1/2: voltage-dependent anion-selective channel protein 1/2
IP3R3: inositol 1,4,5-trisphosphate receptor type 3
FBXL2: F-box protein FBXL2
MITOL: mitochondrial ubiquitin ligase
PINK1: PTEN-induced putative kinase protein 1
TG2: transglutaminase type 2
PDK4: pyruvate dehydrogenase kinase 4
Miro: mitochondrial Rho
PIGBOS: PIGB opposite strand
MICOS: mitochondrial contact site and cristae organizing system
ERMCS: ER-mitochondria contact sites
CLCC1: chloride channel CLIC-like protein 1
ORP5/8: oxysterol-binding protein (OSBP)-related proteins 5/8
PTPIP51: protein tyrosine phosphatase interacting protein-51
VAPB: vesicle-associated membrane protein-associated protein B
Rmdn3: regulator of microtubule dynamics protein 3
PDZD8: PDZ domain-containing protein 8
APP: amyloid-beta precursor protein
SNCA: synuclein-α
Sig-1R: sigma-1 receptor
FTD: frontotemporal dementia
TDP43: TAR DNA-binding protein 43
RAB7: Ras-related protein Rab 7
TBC1D15: TBC1 domain family member 15
FIS1: fission 1 protein
MDVs: mitochondria-derived vesicles
Pex11: Peroxisomal membrane protein 11
ACBD2/ECI2: Acyl-coenzyme A-binding domain 2/ECI2 isoform A.
